# Healing and Antisecretory Effects of Aqueous Extract of* Eremomastax speciosa* (Acanthaceae) on Unhealed Gastric Ulcers

**DOI:** 10.1155/2017/1924320

**Published:** 2017-10-19

**Authors:** A. P. Amang, C. Mezui, G. T. Siwe, J. Emakoua, G. Mbah, E. Z. Nkwengoua, G. E. Enow-Orock, P. V. Tan

**Affiliations:** ^1^Department of Biological Sciences, Faculty of Science, University of Maroua, P.O. Box 814, Maroua, Cameroon; ^2^Department of Biological Sciences, Higher Teachers' Training College, University of Yaoundé I, P.O. Box 047, Yaoundé, Cameroon; ^3^Department of Animal Biology & Physiology, Faculty of Science, University of Yaoundé I, P.O. Box 812, Yaoundé, Cameroon; ^4^Department of Organic Chemistry, Faculty of Science, University of Yaoundé I, P.O. Box 812, Yaoundé, Cameroon; ^5^Department of Biomedical Sciences, Faculty of Health Sciences, University of Buea, P.O. Box 63, Buea, Cameroon

## Abstract

**Objective:**

This work investigated the healing and antisecretory effects of the aqueous extract of* Eremomastax speciosa* on “unhealed gastric ulcers” associated with gastric acid hypersecretion.

**Materials and Methods:**

“Unhealed gastric ulcers” were induced using indomethacin following the establishment of acetic-acid-induced chronic gastric ulcers. The extract (200 and 400 mg/kg, per os) was administered concomitantly with indomethacin (1 mg/kg, subcutaneously). The effects of the extract on both basal and histamine-stimulated gastric acid secretion were determined. Mucus secretion and oxidative stress parameters were measured, and histological assessment of ulcer healing was carried out.

**Results:**

The extract significantly promoted the healing process in rats subjected to “unhealed gastric ulcers” (82.4–88.5% healing rates). Treatment with the extract significantly reduced the basal (25.95–49.51% reduction rates) and histamine-stimulated (24.25–47.41%) acid secretions. The healing effect of the extract was associated with a significant (*p* < 0.05) increase of mucus secretion and concentrations of antioxidant enzymes compared with the controls. The extract at the highest dose showed normalization of the mucosa, without glandular destruction and with the disappearance of fibrosis and lymphocyte infiltration.

**Conclusion:**

The abilities of the extract to increase mucus secretion, to reinforce antioxidant status, and to inhibit acid secretion would be some of the mechanisms by which this extract would accelerate the healing process in “unhealed gastric ulcers.”

## 1. Introduction

Nonsteroidal Anti-Inflammatory Drugs (NSAIDs) are among the most commonly used drugs in the world. In the United States, approximately 70 million prescriptions are written each year, while in Europe these medications represent more than 7.7% of all prescriptions [1, 2]. The frequency of the prescriptions may further increase because of the aging of the population and the probable widening of their use in cancerous and neurological pathology [3, 4]. In fact, more than 90% of prescriptions for NSAIDs are made to patients aged above 65 years. The major problem with the use of these drugs is their involvement in gastric mucosal injury (by inhibition of cyclooxygenases 1 and 2 (COX-1 and COX-2)), oxidative stress, and changes in epithelial cell proliferation/apoptosis balance [5]. NSAIDs delay the healing of gastric ulcers, and this delay is associated with complications such as bleeding, perforation, and, in some cases, death [6]. Globally, ulcerous complication risks are three- to fivefold higher in patients under NSAIDs treatment. Mortality related to these complications is about 5 to 10% for hemorrhagic complications and about 20% for perforations [7].

To counteract the deleterious actions of NSAIDs on the gastrointestinal tract, potentially less toxic NSAIDs (COX-2 inhibitors) have been introduced. In spite of the use of these COX-2 inhibitors, the treatment of gastric ulcers associated with NSAIDs consumption is performed using proton pump inhibitors (PPIs) [8]. Several preclinical and clinical trials demonstrated that PPIs are highly effective in promoting the healing of gastric damage induced by NSAIDs, even in the presence of continued NSAIDs administration [9]. Thus, the control of gastric acid secretion represents a cornerstone for the promotion of ulcer healing [10].

However, treatment using these PPIs presents many adverse effects. For example, the adverse effects attributed to omeprazole, which is the most prescribed PPI, include headaches, diarrhea, abdominal pain, nausea, sleep deprivation [11], pneumonia [12], osteoporosis-related fractures, acute tubule-interstitial nephritis, inflammation of the kidneys, and fundic gland polyposis [13, 14]. This multitude of side effects associated with the most prescribed PPI emphasizes the current need to intensify the search for local medicinal plants with high therapeutic potency and lower toxicity. More than 100 Cameroonian medicinal plants have been cited for the treatment of complaints symptomatic of peptic ulcer disease [15]. Although* Eremomastax speciosa* is not cited in the available literature for its use against gastric complications in folklore medicine, personal information obtained from local traditional healers suggested the possible antiulcer potential of the plant. Thus, in a preliminary study, the water extract of* E. speciosa* significantly inhibited the formation of gastric lesions induced by HCl/ethanol and pylorus ligation [16]. Subsequent studies demonstrated the cytoprotective and antioxidant activities of the methanolic extract against various experimental ulcer models [17]. Other works demonstrated that the antisecretory action of the aqueous extract occurs through a mechanism common to both antihistaminic and anticholinergic pathways [18]. Amang et al. [19] have shown that, in addition to its prophylactic properties, the extract possesses healing actions comparable to ranitidine on chronic gastric ulcers. Nevertheless, these results do not provide any indication of the ability of the extract to heal well-established chronic gastric ulcers associated with repeated NSAIDs intake. Indeed, many elderly patients suffering from chronic gastric ulcers associate their therapy with the use of NSAIDs to relieve the chronic pains linked to other age-related ailments such as arthritis. This causes a delay in the healing of chronic gastric ulcers which are referred to as “unhealed gastric ulcers” [20]. On the other hand, hypersecretion of gastric acid by many patients also inhibits the healing process of gastric ulcers. Thus, in the present work, the healing and antisecretory effects of the aqueous extract of* E speciosa* were evaluated on “unhealed gastric ulcers” associated with gastric acid hypersecretion stimulated by histamine, in addition to the duration of its antisecretory effect. The effects were compared with that of sucralfate, a nonantisecretory mucosal protective agent which has been experimentally shown to speed up gastric ulcer healing.

## 2. Materials and Methods

### 2.1. Materials

#### 2.1.1. Plant Material

The fresh leaves of* E. speciosa* were collected in April 2015, at 6 a.m., in Yaoundé, Center Region of Cameroon. Botanical identification was done at the National Herbarium, Yaoundé, by Paul Mezili, by comparison with existing herbarium specimen number HNC/136984.

#### 2.1.2. Experimental Animals

Male albino Wistar rats aged 12-13 weeks, weighing between 150 and 200 g, were used for the experiments. The animals were raised in the Animal House of the Animal Physiology Laboratory, Faculty of Science, University of Yaoundé I. They were kept at room temperature under natural day/night cycles. They were fed with a standard laboratory diet (supplied by SPC Ltd, Bafoussam, Cameroon) and given tap water ad libitum. Prior authorization for the use of laboratory animals in this study was obtained from the Cameroon National Ethics Committee (registration number: FWA-IRB00001954), which permits, among other procedures, the use of ether anesthesia for animal research. Otherwise, the use, handling, and care of animals were done in adherence to the European Convention for the Protection of Vertebrate Animals Used for Experimental and Other Purposes (ETS-123), with particular attention to Part III, articles 7, 8, and 9 [21].

### 2.2. Methods

#### 2.2.1. Preparation of the Plant Extract

The leaves were chopped and quickly dried under the shade to prevent them from getting moldy and then ground using a mechanical grinder to obtain a fine powder. 400 g of the powder was extracted by infusion in 4 liters of boiled water for 15 minutes. After filtration through Whatman filter paper No. 3, the filtrate was evaporated using a Raven ventilation oven (Jencons PLS, UK). The brownish solid obtained (99.7 g, 24.9% yield) was stored at 4°C. The extract was dissolved in distilled water which was used as the vehicle.

#### 2.2.2. Induction of Gastric Ulcers

*Induction of “Unhealed Gastric Ulcers” and Basal Acid Secretion*. The method described by Pillai and Santhakumari [22] was used, with slight modifications in accordance with the procedure described by Wang et al. [23]. Briefly, laparotomy was performed under ether anesthesia on experimental rats after 24-hour fast. Fifty microliters of 30% glacial acetic acid was injected into the wall of the stomach corpus at the region of the lesser curvature and the stomach wall was wiped using cotton wool soaked in a 9‰ NaCl solution. The abdominal incisions were stitched up and feeding was resumed. A disinfectant (Betadine®) was applied daily to avoid infection. Four days after the operation, a control group (4th-day control) was sacrificed using ether, and their stomachs were opened in order to establish the degree of ulceration prior to the onset of treatment.

Five days after the acetic acid injection, indomethacin suspended in saline solution was given once daily to all the remaining rats (1 mg/kg, s.c.) for 2 weeks in order to establish “unhealed gastric ulcers.” The extract (200 and 400 mg/kg), sucralfate (50 mg/kg), or vehicle (1 ml/200 g b.w.) alone was given per os (p.o.) once daily 0.5 hours after indomethacin treatment for 2 weeks. On day 13 of treatment (24 hours before the final administration of vehicle, extract, or sucralfate), the animals were deprived of food but allowed free access to water. 0.5 or 12 hours after the final administration (day 14), basal acid secretion was studied by the pylorus ligation technique described by Shay et al. [24]. Six hours later, the rats were sacrificed using ether and the gastric contents produced by each animal were collected and centrifuged and the supernatant was measured. Ulcer areas and gastric mucus production were measured. Ulcer healing rates were calculated by comparing the ulcer status of extract- and sucralfate-treated rats with those of the vehicle controls. Spontaneous healing was evaluated by comparing the vehicle controls with the 4th-day controls. Gastric tissue samples were collected and prepared for the measurement of different oxidative stress parameters. The stomach ulcerated portions were fixed and stored in 10% formaldehyde awaiting histological studies.

*Induction of “Unhealed Gastric Ulcers” and Histamine-Induced Gastric Hypersecretion*. The same protocol described above was performed but with slight modifications. Following the establishment of “unhealed gastric ulcers,” 0.5 or 12 hours after the final administration of vehicle, sucralfate, or extract, pylorus ligation was performed in the rats. Histamine-induced gastric acid hypersecretion was studied by injecting histamine (2.5 mg/kg, s.c.) in all the remaining rats 1 hour after pylorus ligature. Four hours later, the animals were sacrificed and submitted to the same procedures as described above.

#### 2.2.3. Measurement of Mucus Production

The mucus covering each stomach was gently scraped using a glass slide and the mucus was weighed carefully using a sensitive digital electronic balance. The same experimenter performed this exercise each time [25].

#### 2.2.4. Measurement of Gastric Acidity

The gastric contents were collected and centrifuged at 4000 rpm for 10 min, to remove residual debris. The volume of the gastric juice was measured using a graduated test tube. 1 ml of centrifuged gastric juice was used to determine the hydrogen ion concentration by pH-metric titration against NaOH solution (0.1 N) using a digital pH meter. The acid content was expressed as mEq/l [16].

#### 2.2.5. Preparation of Histological Sections

Sections of stomach walls were made perpendicular to the surface of each ulcer crater. Sections of the normal stomach were also made for comparison. Hematoxylin and eosin stains of stomach sections were then prepared following standard histological procedures described by Bayelet-Vincent [26] and the sections were observed microscopically.

#### 2.2.6. Measurement of* In Vivo* Antioxidant Capacity

Oxidative stress parameters were measured in gastric tissue samples. Superoxide dismutase (SOD) activity was measured using a standard method [27] and expressed in U/mg of protein. Catalase (CAT) was determined [28] and expressed as *μ*mol of H_2_O_2_/min/mg of protein. Reduced glutathione (GSH) was measured based on the reaction between 2,2-dithio-5,5-dibenzoic acid and the thiol (SH) groups of glutathione to yield a complex whose absorbance was read at 412 nm [29]. The glutathione concentration was calculated using the molar extinction coefficient *ε* = 13600 cm/mol and the results are expressed in nmol/g of tissue. Lipid peroxidation was assessed by measuring the levels of malondialdehyde (MDA) in gastric tissue samples [30]. Quantification of MDA was done using an extinction coefficient of 1.56 × 10^5^ M^−1^ cm^−1^ and expressed as *μ*mol of MDA/g of wet stomach tissue. Tissue protein was measured using the Biuret method of protein assay [31].

#### 2.2.7. Statistical Analysis

Values in tables are given as arithmetic means ± standard error of the mean (SEM). The significance of differences between groups was analyzed by means of Analysis of Variance (ANOVA) followed by Student-Newman-Keuls comparison tests using GraphPad Prism 5.03 software. All the differences were considered significant at *p* < 0.05.

## 3. Results

### 3.1. Ulcer Healing

**Figure 1:**
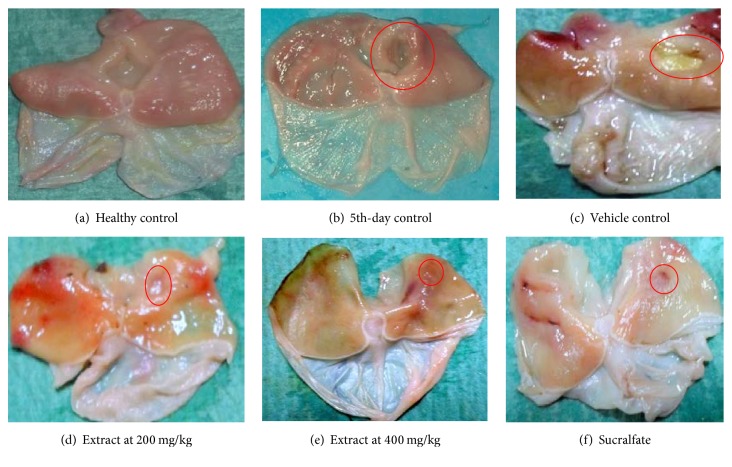
Macroscopic aspects of “unhealed gastric ulcers” in rats. The red circle indicates the position of an ulcer. (a) Control nonulcerated rats (showing normal healthy mucosa). (b) Ulcerated rats sacrificed on day 5 after ulcer induction (showing well-defined ulcer craters, with raised, inflamed ulcer margins). (c) Ulcerated rats that were given the vehicle and indomethacin for 2 weeks (ulcer craters are reduced due to autohealing). (d–f) Ulcerated rats that were given the extract or sucralfate plus indomethacin for 2 weeks (with significant disappearance of ulcer craters especially at (e) 400 mg/kg extract).

The macroscopic aspects of the stomachs subjected to “unhealed gastric ulcers” are shown in Figure 1. The stomachs of the 4th-day controls showed deep and wide craters (Figure 1(b)) representing an ulcerated area of 61.20 ± 3.61 mm^2^ (9.07% of glandular area). In the control rats (vehicle control) that were given the vehicle concomitantly with indomethacin during the two weeks following the establishment of ulcers, the ulcer area dropped to 39.20 ± 7.71 mm^2^ (pylorus ligation) or 42.50 ± 5.29 mm^2^ (histamine + pylorus ligation) indicating autohealing of 35.95% and 30.56%, respectively. The extract, given concomitantly with indomethacin for 2 weeks, significantly promoted the healing process (Figures 1(d) and 1(e)). In rats subjected to “unhealed gastric ulcers”/pylorus ligature, ulcerated areas reduced from 39.20 ± 7.71 mm^2^ in vehicle controls to 6.50 ± 0.39 mm^2^ and 4.50 ± 0.22 mm^2^ for the extract doses of 200 and 400 mg/kg, respectively, representing healing rates of 83.4 and 88.5% (Table 1). Similar results were obtained with extract (200 and 400 mg/kg) in rats subjected to “unhealed gastric ulcers”/pylorus ligature/histamine injection (7.50 ± 0.39 mm^2^ and 6.30 ± 0.37 mm^2^), with healing rates of 82.35 and 85.17%, respectively (Table 2). The healing process was associated in all the tested models with a significant increase in mucus production compared with the vehicle controls.

**Figure 2:**
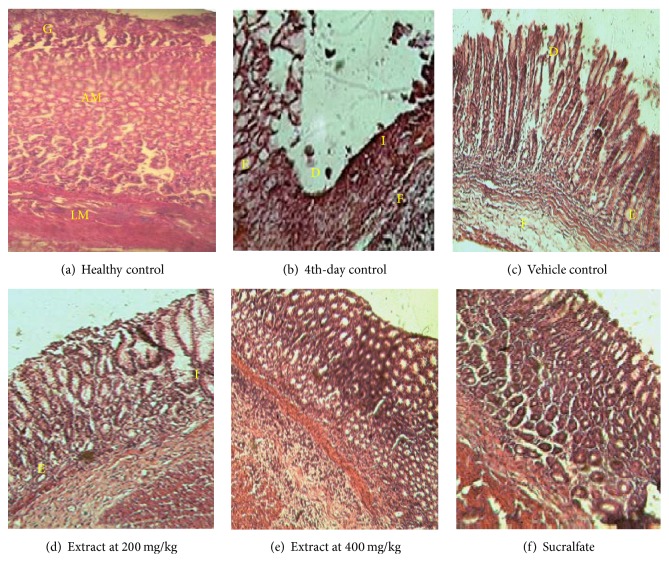
Histological presentation of stomach sections of “unhealed gastric ulcers” (H&E, ×100). (a) Stomach section of control nonulcerated rats with intact gastric glands (G), annular muscles (AM), and longitudinal muscles (LM). (b) Section in ulcerated rats sacrificed on day 5 after ulcer induction, showing an ulcer crater with complete destruction of glands at the center (D). The desquamated glandular detritus can be seen lying in the lumen, with pockets of edema (E) and fibrosis (F) and leucocyte infiltration (I). (c) Ulcerated rats that were given the vehicle and indomethacin for 2 weeks; the process of autohealing is depicted by glandular regrowth and reduced presence of desquamated material. (d–f) Ulcerated rats that were given the extract or sucralfate plus indomethacin for 2 weeks; sections show significant reestablishment of gastric glands at 200 mg/g of extract and advanced reepithelialization and compact glands for extract (400 mg/kg) and sucralfate.

The histological presentation of “unhealed gastric ulcers” is shown in Figure 2. The stomach sections of healthy rats showed normal gastric mucosal glands (Figure 2(a)). In the 4th-day control, histological observation showed superficial loss of mucosal substance. Many glands had sloughed glandular epithelial cells lying loosely in the gland lumen and many inflammatory cells could be seen in the interstitial tissue. Fibrosis, sclerosis, and edema were also observed (Figure 2(b)). In the vehicle control, ulcer sections showed persistence of mucosa destruction, sclerotic block, and leukocyte infiltration with regression of edema (Figure 2(c)). In the extract-treated rats (200 mg/kg), stomach sections showed amelioration of gastric tissues, with the disappearance of fibrosis but with persistent edema (Figure 2(d)). Treatment with extract (400 mg/kg) and sucralfate showed normalization of the mucosa, without glandular destruction and with disappearance of fibrosis and lymphocyte infiltration (Figures 2(e) and 2(f)).

Tables 3 and 4 show, respectively, the results obtained when the basal or histamine-stimulated gastric secretions in rats with “unhealed ulcers” were measured 0.5 hr after the final administration of the extract. In the vehicle control, acidity stimulated by histamine (88.30 ± 0.92 mEq/L) was higher than basal acidity (64.23 ± 4.29 mEq/L), corresponding to an increase of 27.26%. Treatment with the extract (200 and 400 mg/kg) significantly reduced the basal (42.39 ± 3.32 and 32.43 ± 2.47 mEq/L) and histamine-stimulated (59.00 ± 4.08 and 46.44 ± 3.29 mEq/L) acid secretions. The extract-induced reductions in acid secretion were accompanied by significant reductions in volumes of gastric juice and gastric pH in both experimental models.

The effects of* E. speciosa* on basal and histamine-induced gastric acid secretions 12 hr after final extract administration are shown in Tables 5 and 6. The vehicle controls exhibited similar results for acid secretion compared with those observed 0.5 hr after the final administration of the vehicle. On the other hand, extract-treated groups showed lower inhibition of acid secretion compared to that observed 0.5 hr after the final administration of the extract. Thirty minutes after the final administration of the extract, the reduction rate of the basal secretion of acid dropped from 34.00 and 49.51%, at the doses of 200 and 400 mg/kg, respectively, to 25.95 and 32.97% twelve hours after the final administration of the extract at the same doses. In the same way, the extract effect on histamine-stimulated acid secretion dropped in a time-dependent manner.

### 3.2. Antioxidant Activity

Tables 7 and 8 show the* in vivo* antioxidant effects of* E. speciosa* in rats subjected to “unhealed gastric ulcers.” The levels of superoxide dismutase (SOD), catalase (CAT), and reduced glutathione (GSH) decreased, and malondialdehyde (MDA) increased, in the vehicle control compared with normal rats. In the two experimental methods, extract administration significantly increased concentrations of SOD, CAT, and GSH compared with the vehicle controls. On the contrary, the levels of MDA significantly decreased in extract-treated rats compared with the vehicle controls. However, these levels of oxidative stress parameters did not return to those observed in normal rats.

## 4. Discussion

Previous studies revealed the prophylactic and healing activities of the aqueous extract of* E. speciosa* on gastric ulcers [17–19]. In the current work, the healing and antisecretory effects of this extract were evaluated on “unhealed gastric ulcers” as well as the duration of the antisecretory effect. The results of the present work show that the extract, given concomitantly with indomethacin for 2 weeks, significantly promoted the healing of acetic-acid-induced gastric ulcers. The healing process of the ulcers was associated with a significant increase in mucus production compared with the vehicle control.

The experimental model of acetic-acid-induced chronic ulcers easily and reliably produces round, deep ulcers in the stomach that highly resemble human ulcers in terms of pathology and healing [20]. These ulcers are mainly due to the corrosive action of acetic acid. In addition, the pain generated by laparotomy constitutes a source of stress which leads to an increase of gastric acid secretion. This increase involves back-diffusion of the H^+^ ions through the channels in the mucus layer, towards the internal layers of the gastric membrane. This results in tissue necrosis which triggers the release of arachidonic acid metabolites and attracts leucocytes (polynuclear neutrophils and macrophages) leading to the transformation of superficial injury into deeper mucosal lesions and to inactivation of growth factors essential for mucosal integrity and repair [32, 33]. This explains the high degree of ulceration (61.20 ± 3.61 mm^2^) observed in ulcerated rats (Control 1) sacrificed 4 days after acetic acid ulcer induction.

Previous work [19] showed that, in the ulcerated control animals (control 2) that were given the vehicle alone during the two-week period of treatment, ulcer craters reduced in size by the process of autohealing (44.98%). This autohealing is due to the mucosal damage which constructively stimulates the secretion of growth factors in the adjacent mucosa and ulcer bed [34]. Concomitant administration of the vehicle and indomethacin during the same period reduced the degree of autohealing to 35.95% and 30.56%, with and without histamine stimulation, respectively. In humans and experimental models, peptic ulcer healing is delayed by NSAIDs [34]. Wang et al. [35] demonstrated that repeated administration of indomethacin markedly prevents spontaneous healing of acetic-acid-induced ulcers. Brzozowski et al. [36] reported that indomethacin-induced delayed healing was due to suppression of endogenous prostaglandins (PGE) and excessive cytokine expression and release. The resulting decrease in endogenous prostaglandins (PGE) secretion weakens the mucosal defensive mechanism. As a result, even basal gastric acid secretions might attack the weakened ulcerated area, resulting in delayed ulcer healing [20]. Taken together, the increase in proinflammatory cytokines (TNF-*α*, interleukin-1*β*) and the impairment of growth factor biosynthesis (bFGF, HGF, and EGF) might contribute to the mechanism underlying “unhealed gastric ulcers.”

In this work,* E. speciosa* extract (200 and 400 mg/kg) significantly prevented the delayed ulcer healing in response to indomethacin without histamine (83.4 and 88.5% healing rate, resp.) and with histamine (82.35 and 85.17% healing rate, resp.) stimulation. The healing process of the extract was associated in all the tested models with a significant increase in mucus production compared with the vehicle control. The importance of increased mucus strength and quantity in protecting the regenerating gastric epithelium is well known [5, 37]. Indeed, mucus ensures double protection: physical protection while acting as a lubricant for the gastric mucosa by preventing the direct contact between gastric juice and gastric epithelium, thus favoring the healing process, and chemical protection acting against the proteolytic and acid properties of gastric juice by sequestering bicarbonate, creating a pH gradient between the gastric juice and the gastric epithelium [38].

In previous work, Amang et al. [19] demonstrated that the inhibitory effect of* E. speciosa* extract against HCl/ethanol-induced gastric ulcers was suppressed when the rats were pretreated with indomethacin, and it was interpreted that the extract was acting through the intermediary of endogenous PGE. This interpretation was supported by the significant increase of mucus production in extract-treated animals compared with the vehicle control. Endogenous PGs play an essential protective role in the stomach by stimulating the synthesis and secretion of mucus and bicarbonate, increasing mucosal blood flow, and promoting epithelial proliferation [39, 40]. Okabe and Amagase [41] showed that prostaglandin analogs (sucralfate) also significantly enhance healing of acetic-acid-induced ulcers. Wang et al. [35] reported that the delayed healing can be prevented using exogenously administered PGE_2_. Thus, the stimulation of mucus secretion mediated by PGE could partly explain the mechanism by which the extract prevents the delayed healing caused by indomethacin in acetic-acid-induced ulcers

The pathogenesis of “unhealed gastric ulcers” also involves the generation of reactive oxygen species (ROS) [42] due to the combined actions of laparotomy [43], acetic acid injection, and indomethacin administration [44]. Taken together, these mechanisms cause ROS hyperproduction whose involvement in the physiopathology of gastrointestinal inflammation and gastric ulcer is well known [45]. Overproduction of ROS is known to be one of the major pathogenic factors that directly results in oxidative damage, including lipid peroxidation, protein oxidation, and DNA damage, which can lead to cell death [46]. MDA, a product of polyunsaturated fatty acids peroxidation and related esters, is a suitable index of oxidative tissue damage [47]. Furthermore, the increase of MDA level is generally associated with an impairment of antioxidative defense mechanisms (SOD, CAT, and GSH) [42]. Thus, high levels of MDA were associated with low levels of SOD, CAT, and GSH in the ulcerated untreated control rats compared with normal rats. The role of these factors (SOD, CAT, and GSH) in the defense against oxidative stress is well known. SOD catalyzes the dismutation of superoxide radical anion (O_2_^•−^) into less noxious hydrogen peroxide (H_2_O_2_), which is further degraded by catalase or glutathione peroxidase. Catalase enzyme accelerates the degradation of H_2_O_2_ into water and oxygen [48]. The second pathway of H_2_O_2_ metabolism depends on the activity of glutathione peroxidase (GPx) and cooperating glutathione reductase. The reduction of H_2_O_2_ into water by GPx is accompanied by the conversion of glutathione from the reduced form into the oxidized form [49–51]. The significant increases of SOD, CAT, and GSH concentrations in extract-treated rats are evidence of their implication in the enhancement of the antioxidant status and consequently the ulcer healing mechanism. Previous work [18] reported the presence of alkaloids, flavonoids, triterpenoids, phenols, and tannins in the aqueous extract of* E. speciosa*. These phytochemical compounds have well-known antioxidant activity and have been associated with gastric mucosal protection [52–57].

Gastric acid secretion is significantly involved in the delayed healing of gastric ulcers, and the control of gastric acid secretion represents a cornerstone for the promotion of ulcer healing [10]. There is also evidence that NSAIDs can interfere with ulcer healing by both acid-dependent and acid-independent mechanisms [34]. PPIs such as omeprazole have been shown to reverse NSAID-induced deleterious effects on gastric ulcer healing even in the presence of continued NSAID administration. These effects occur through the activation of acid-dependent mechanisms [9] and marked inhibition of acid secretion, involved in the regulation of mucosal cell proliferation in the ulcer margin [58–60]. Other reports have suggested that several growth factors (e.g., HGF) are involved in the ulcer healing effects of PPIs in indomethacin-induced gastric damage [61]. Expression of EGF has been found to increase in the gastric mucosa of mice with indomethacin-induced injury and to be further enhanced by omeprazole [62]. In previous work, Amang et al. [18] showed that the aqueous extract of* E. speciosa* has antisecretory effects which involve a mechanism common to both cholinergic and histaminergic pathways. Consequently, the observed significant drop of basal and stimulated (histamine) acid secretion by the extract in the present work suggests the implication of its antisecretory effects in the healing process.

The extract, given repeatedly for 2 weeks, significantly inhibited both basal and histamine-stimulated gastric secretion in rats with “unhealed gastric ulcers” up to 12 hours after the last administration. Wang et al. [23] reported that, in rats with ulcers, 24 hours after the final administration of omeprazole for 4 weeks, both basal and histamine-stimulated gastric secretions were significantly inhibited. They suggested that the preventive effect of omeprazole on delayed ulcer healing is causally related to its long-lasting antisecretory effect. In a similar way, the preventive effect of* E. speciosa* extract on the indomethacin-induced delay of chronic ulcer healing may occur by the same mechanism.

Histological sections of rat stomachs illustrate the damage caused by acetic acid and indomethacin as well as the healing process induced by the extract. In general, following ulcerative injury, ulcer healing is initiated by the formation of the “healing zone.” At this stage, inflammatory infiltration occurs close to the necrotic tissue and ulcer crater. In response to growth factors, the ulcer margin is formed by cells adjacent to the margin and granulation tissue develops at the ulcer base. During healing, the granulation tissue undergoes continuous remodeling, whereby the inflammatory cells that appeared in the early phase of healing are replaced by fibroblasts and microvessels in the late healing phase [63]. The major stimuli for cell migration and ulcer reepithelialization are mediated by growth factors which are produced by platelets, injured tissue, and macrophages. The migration of epithelial cells from the ulcer margin, to restore the continuity of the epithelial lining, is essential for ulcer healing since it generates a barrier protecting the granulation tissue from any mechanical and chemical damage [64]. The aqueous extract of* E. speciosa* accelerated the healing of “unhealed gastric ulcers” following the combined action of several mechanisms which involve the increases of mucus production and antioxidant levels on the one hand and the decrease of gastric acid secretion on the other hand. All these factors taken together can enhance angiogenesis, cellular proliferation, cellular migration, and granulation tissue maturation. The results of the present study suggest that* E. speciosa* extract can be a useful resource for the production of a standardized antiulcer product, especially given that toxicological studies have demonstrated the innocuousness of this plant extract [65].

*Study Limitations*. This study did not provide information on the identification of the active components of* E. speciosa* extract. Thus, a detailed discussion of the exact mechanism of antisecretion and healing action of the extract could not be given.

Second, data for sucralfate antisecretory effects against histamine-induced hypersecretion are not provided for detailed discussion of results in Tables 4, 5, 6, and 8.

## 5. Conclusion

The abilities of* E. speciosa* extract to increase mucus production, to reinforce the antioxidant status, and to inhibit acid secretion by a mechanism involving both cholinergic and histaminic pathways constitute possible ways by which the extract accelerates the healing of “unhealed gastric ulcers.” This healing process might be reinforced by the long-lasting antisecretory effect of the extract. Thus, the use of this plant extract could be recommended for the management of chronic gastric ulcers for patients who are concomitantly taking NSAIDs to treat other ailments.

## Figures and Tables

**Table 1:** Effect of *E. speciosa* on “unhealed gastric ulcers” in pylorus-ligated rats.

Treatment	Dose (mg/kg)	*N*	Ulcerated area (mm^2^)	% ulcerated area	% healing	Mucus production (mg)
Control 1	—	5	61.20 ± 3.61	9.07	—	78.68 ± 4.00
Control 2	—	5	39.20 ± 7.71	5.81	35.95	50.50 ± 1.47
*E. speciosa*	200	5	6.50 ± 0.39^*∗∗∗*^	0.96	83.42	80.25 ± 5.12^*∗∗*^
*E. speciosa*	400	5	4.50 ± 0.22^*∗∗∗*^	0.67	88.52	81.00 ± 3.42^*∗∗*^
Sucralfate	50	5	6.25 ± 0.80^*∗∗∗*^	0.93	84.06	76.50 ± 3.29^*∗∗*^

*N*: number of rats. The values are expressed as mean ± SEM; ^*∗∗*^ *p* < 0.01; ^*∗∗∗*^ *p* < 0.001; statistically different compared to Control 2. Control 1: ulcerated rats sacrificed 4 days after acetic acid ulcer induction. Control 2: ulcerated rats given indomethacin alone for 14 days following ulcer induction.

**Table 2:** Effect of *E. speciosa* on “unhealed gastric ulcers” in pylorus-ligated rats treated with histamine.

Treatment	Dose (mg/kg)	*N*	Ulcerated area (mm^2^)	% ulcerated area	% healing	Mucus production (mg)
Control 1	—	5	61.20 ± 3.61	9.07	—	78.68 ± 4.00
Control 2	—	5	42.50 ± 5.29	6.30	30.56	49.25 ± 2.37
*E. speciosa*	200	5	7.50 ± 0.39^*∗∗∗*^	1.11	82.35	70.00 ± 0.63^*∗∗∗*^
*E. speciosa*	400	5	6.30 ± 0.37^*∗∗∗*^	0.93	85.17	80.25 ± 3.77^*∗∗∗*^

*N*: number of rats. The values are expressed as mean ± SEM; ^*∗∗∗*^ *p* < 0.001; statistically different compared to Control 2. Control 1: ulcerated rats sacrificed 4 days after acetic acid ulcer induction. Control 2: ulcerated rats given indomethacin alone for 14 days following ulcer induction.

**Table 3:** Effect of *E. speciosa* on basal gastric acid secretion (after 0.5 hr) in rats subjected to “unhealed gastric ulcers.”

Treatment	Dose (mg/kg)	*N*	Volume of gastric juice (ml)	Gastric pH	Gastric acidity (mEq/l)	% reduction of gastric acidity
Control	—	5	6.57 ± 0.28	2.43 ± 0.15	64.23 ± 4.29	—
*E. speciosa*	200	5	4.28 ± 0.21^*∗∗∗*^	4.00 ± 0.45^*∗∗*^	42.39 ± 3.32^*∗∗∗*^	34.00
*E. speciosa*	400	5	3.56 ± 0.25^*∗∗∗*^	4,27 ± 0,28^*∗∗*^	32.43 ± 2.47^*∗∗∗*^	49.51
Sucralfate	50	5	2.46 ± 0.23^*∗∗∗*^	3.01 ± 0.27	41.59 ± 2.52^*∗∗∗*^	35.25

*N*: number of rats. The values are expressed as mean ± SEM; ^*∗∗*^ *p* < 0.01; ^*∗∗∗*^ *p* < 0.001; statistically different compared to control.

**Table 4:** Effect of *E. speciosa* on histamine-induced gastric acid hypersecretion (after 0.5 hr) in rats subjected to “unhealed gastric ulcers.”

Treatment	Dose (mg/kg)	*N*	Volume of gastric juice (ml)	Gastric pH	Gastric acidity (mEq/l)	% reduction of gastric acidity
Control 1	—	5	2.30 ± 0.26	2.56 ± 0.20	88.30 ± 0.92	
*E. speciosa*	200	5	3.74 ± 0.04^*∗∗*^	3.59 ± 0.15^*∗∗*^	59.00 ± 4.08^*∗∗∗*^	33.18
*E. speciosa*	400	5	4.51 ± 0.32^*∗∗∗*^	3.77 ± 0.20^*∗∗*^	46.44 ± 3.29^*∗∗∗*^	47.41

*N*: number of rats. The values are expressed as mean ± SEM; ^*∗∗*^ *p* < 0.01; ^*∗∗∗*^ *p* < 0.001; statistically different compared to control.

**Table 5:** Effect of *E. speciosa* on basal gastric acid secretion (after 12 hr) in rats subjected to “unhealed gastric ulcers.”

Treatment	Dose (mg/kg)	*N*	Volume of gastric juice (ml)	Gastric pH	Gastric acidity (mEq/l)	% reduction of gastric acidity
Control	—	5	3.78 ± 0.11	3.07 ± 0.26	64.28 ± 4.44	—
*E. speciosa*	200	5	4.79 ± 0.32^*∗*^	3.85 ± 0.13^*∗*^	47.60 ± 2.49^*∗∗∗*^	25.95
*E. speciosa*	400	5	4.24 ± 0.22	4.37 ± 0.19^*∗∗*^	43.09 ± 3.25^*∗∗∗*^	32.97

*N* = 5 rats per treatment. The values are expressed as mean ± SEM; ^*∗*^ *p* < 0.05; ^*∗∗*^ *p* < 0.01; ^*∗∗∗*^ *p* < 0.001; statistically different compared to control.

**Table 6:** Effect of *E. speciosa* on histamine-induced gastric acid hypersecretion (after 12 hr) in rats subjected to “unhealed gastric ulcers.”

Treatment	Dose (mg/kg)	*N*	Volume of gastric juice (ml)	Gastric pH	Gastric acidity (mEq/l)	% reduction of gastric acidity
Control	—	5	5.22 ± 0.26	3.42 ± 0.24	89.60 ± 3.24	—
*E. speciosa*	200	5	2.64 ± 0.11^*∗∗∗*^	4.04 ± 0.29	67.60 ± 5.49^*∗∗∗*^	24.55
*E. speciosa*	400	5	4.10 ± 0.13^*∗∗*^	4.88 ± 0.41^*∗*^	63.06 ± 5.25^*∗∗∗*^	29.62

*N* = 5 rats per treatment. The values are expressed as mean ± SEM; ^*∗*^ *p* < 0.05;^*∗∗*^ *p* < 0.01; ^*∗∗∗*^ *p* < 0.001; statistically different compared to control.

**Table 7:** Antioxidant effects of *E. speciosa* in rats subjected to “unhealed gastric ulcers”/pylorus ligature.

Treatment	Dose (mg/kg)	SOD (U/mg protein)	Catalase (*µ*mol H_2_O_2_/min/mg protein)	GSH (mmol/g protein)	Malondialdehyde (pmol/mg protein)
Normal rats	—	7.54 ± 0.16	5.22 ± 0.22	2.61 ± 0.02	1.60 ± 0.07
Control 1	—	4.02 ± 1.53^*∗∗∗*^	0.54 ± 0.03^*∗∗∗*^	0.85 ± 0.80^*∗*^	4.04 ± 0.25^*∗∗∗*^
Control 2	—	4.50 ± 0.37^*∗∗*^	0.65 ± 0.04^*∗∗∗*^	0.96 ± 0.11^*∗*^	4.46 ± 0.37^*∗∗∗*^
*E. speciosa*	200	6.82 ± 0.28^*∗*^	5.83 ± 0.23^ΦΦΦ^	3.24 ± 0.24^ΦΦ^	2.25 ± 0.01^*∗∗*ΦΦ^
*E. speciosa*	400	7.79 ± 0.08^*∗∗*^	7.84 ± 0.28^*∗∗*ΦΦΦ^	4.89 ± 0.18^*∗∗*ΦΦΦ^	1.50 ± 0.06^ΦΦΦ^
Sucralfate	50	7.25 ± 0.32^*∗*^	5.04 ± 0.20^ΦΦΦ^	2.37 ± 0.10^ΦΦ^	2.58 ± 0.34^*∗*ΦΦ^

*N* = 5 rats per treatment. Control 1: ulcerated rats sacrificed 5 days after ulcer induction. The values are expressed as mean ± SEM; ^*∗*^ *p* < 0.05; ^*∗∗*^ *p* < 0.01; ^*∗∗∗*^ *p* < 0.001; statistically different compared to normal rats; ^ΦΦ^ *p* < 0.01; ^ΦΦΦ^ *p* < 0.001; statistically different compared to Control 2.

**Table 8:** Antioxidant effects of *E. speciosa* in rats subjected to “unhealed gastric ulcers”/pylorus ligature/histamine.

Treatment	Dose (mg/kg)	SOD (U/mg protein)	Catalase (*µ*mol H_2_O_2_/min/mg protein)	GSH (mmol/g protein)	Malondialdehyde (pmol/mg protein)
Normal rats	—	7.54 ± 0.16	5.22 ± 0.22	2.61 ± 0.02	1.60 ± 0.07
Control 1	—	4.02 ± 1.53^*∗∗∗*^	0.54 ± 0.03^*∗∗∗*^	0.85 ± 0.80^*∗∗*^	4.04 ± 0.25^*∗∗∗*^
Control 2	—	4.57 ± 0.38^*∗∗*^	0.71 ± 0.04^*∗∗∗*^	0.85 ± 0.20^*∗∗*^	4.24 ± 0.29^*∗∗∗*^
*E. speciosa*	200	5.75 ± 0.33^#**∗**^	4.14 ± 0.25^*∗*ΦΦΦ^	2.69 ± 0.17^ΦΦ^	2.35 ± 0.37^*∗*ΦΦ^
*E. speciosa*	400	7.42 ± 0.19^##**∗∗**^	5.94 ± 0.45^ΦΦΦ^	3.62 ± 0.48^*∗*ΦΦ^	1.46 ± 0.11^ΦΦΦ^

*N* = 5 rats per treatment. Control 1: ulcerated rats sacrificed 5 days after ulcer induction. The values are expressed as mean ± SEM; ^*∗*^ *p* < 0.05; ^*∗∗*^ *p* < 0.01; ^*∗∗∗*^ *p* < 0.001; statistically different compared to normal rats; ^ΦΦΦ^ *p* < 0.01; ^ΦΦΦ^ *p*< 0.001; statistically different compared to Control 2; Control 1 or 4th day control; Control 2 or vehicle control. ^#^ *p* < 0.05 and ^##^ *p* < 0.01 compared with Control 1.
